# Severe traumatic tricuspid regurgitation detected 8 years after chest trauma

**DOI:** 10.1007/s10396-024-01452-w

**Published:** 2024-04-22

**Authors:** Takahiro Nishihara, Yoichi Takaya, Norihisa Toh, Shinsuke Yuasa

**Affiliations:** Department of Cardiovascular Medicine, Dentistry and Pharmaceutical Sciences, Okayama University Graduate School of Medicine, 2-5-1 Shikata-Cho, Kita-Ku, Okayama, 700-8558 Japan

**Fig. 1:**
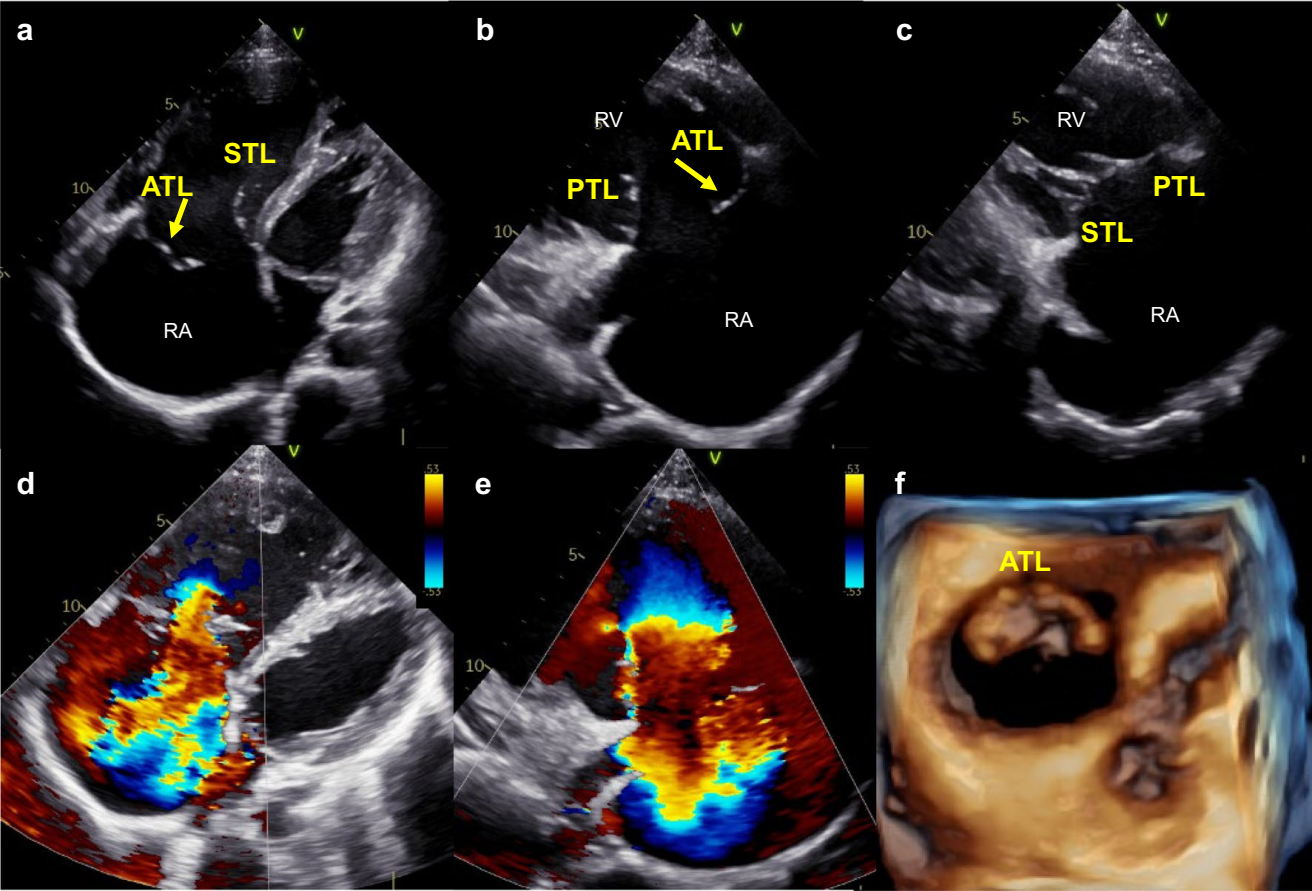
**a** Two-dimensional transthoracic apical four-chamber view showed prolapse of the ATL (arrow) and enlargement of the RA and right ventricle. (**b**, **c**) Two-dimensional transthoracic parasternal right ventricular inflow view showed prolapse of the ATL (arrow) and good coaptation of the STL and PTL. (**d**, **e**) Color flow Doppler showed severe TR. **f** Three-dimensional view from the right atrial aspect showed prolapse of the ATL. RA, right atrium; ATL, anterior tricuspid leaflet; STL, septal tricuspid leaflet; TR, tricuspid regurgitation; PTL, posterior tricuspid leaflet

A healthy 25-year-old man with a grade 3/6 pansystolic murmur in the fourth intercostal space near the lower sternal border presented to our hospital. At 17 years of age, he had been involved in a high-energy moped accident, but he had no symptoms after the event. He also had no symptoms or physical findings, such as systolic positive wave of juggler vein, pulsatile liver, or pretibial edema when he visited our hospital. An electrocardiogram showed complete right bundle branch block. Transthoracic echocardiography showed an enlarged right atrium (RA) and right ventricle with preserved systolic function. Prolapse of the anterior tricuspid leaflet (ATL) without obvious chordae tendineae was detected in the RA (Fig. 1a). The septal tricuspid leaflet (STL) and posterior tricuspid leaflet (PTL) were coapted, but the ATL was not coapted with either the STL or PTL (Fig. 1a-–c). The STL was attached to the apex approximately 13 mm from the mitral valve, but no misalignment of the PTL attachment was detected. Color flow Doppler revealed severe tricuspid regurgitation (TR) (Fig. 1d, e). Three-dimensional transthoracic echocardiography showed prolapse of the whole ATL with a large coaptation defect (Fig. 1f). The brain natriuretic peptide concentration was 142.9 pg/mL. Right heart catheterization showed an elevated mean RA pressure of 10 mmHg and decreased cardiac index of 1.84 L/min/m^2^. The mean pulmonary artery wedge pressure and mean pulmonary artery pressure were 5 mmHg and 11 mmHg, respectively. Traumatic TR was diagnosed based on the patient’s history and echocardiography findings. He is undergoing careful follow-up with consideration of early surgery in the future.

This case demonstrates the necessity of transthoracic echocardiography after blunt chest trauma. The proposed mechanisms of tricuspid injury are severe chest wall compression, deceleration force, and a sudden increase in right ventricular pressure due to its position (1). The causes of traumatic TR include chordal rupture, papillary muscle rupture, and leaflet rupture (2). The diagnosis of traumatic TR may be overlooked because of the slow pathologic progression and asymptomatic condition. Clinical practitioners are less aware of the possibility of TR caused by traumatic chest injury than other causes. This lack of awareness may lead to delayed treatment, resulting in irreversible dilatation of the right atrium and ventricle. The success rate of tricuspid valve repair decreases in such cases (3), and the prognosis is poor (4). For patients with a history of a traffic accident, the possibility of asymptomatic TR should be kept in mind. Echocardiographic screening is recommended considering TR occurrence in such patients.
